# A commentary on ‘Evolutionary patterns and research frontiers in neoadjuvant immunotherapy: a bibliometric analysis’

**DOI:** 10.1097/JS9.0000000000000529

**Published:** 2023-06-20

**Authors:** Kunming Cheng, Yongbin He, Shuqin Gu, Haiyang Wu, Cheng Li

**Affiliations:** a Department of Intensive Care Unit, The Second Affiliated Hospital of Zhengzhou University, Zhengzhou; b School of Sports Medicine and Rehabilitation, Beijing Sport University; c Department of Orthopaedic Surgery, Beijing Jishuitan Hospital, Fourth Clinical College of Peking University; d State Key Laboratory of Toxicology and Medical Countermeasures, Beijing Institute of Pharmacology and Toxicology, Beijing; e Department of Clinical College of Neurology, Neurosurgery and Neurorehabilitation; f Department of Graduate School, Tianjin Medical University, Tianjin, People’s Republic of China; g Duke Molecular Physiology Institute, Duke University School of Medicine; h University of North Carolina at Chapel Hill, Chapel Hill; i Duke Human Vaccine Institute, Duke University Medical Center, Durham, North Carolina, USA; j Center for Musculoskeletal Surgery (CMSC), Charité-Universitätsmedizin Berlin, corporate member of Freie Universität Berlin, Humboldt University of Berlin, and Berlin Institute of Health, Berlin, Germany

*Dear Editor*,

We read with great interest the paper by Jiang *et al.* ^1^, titled ‘Evolutionary patterns and research frontiers in neoadjuvant immunotherapy: a bibliometric analysis’, which is published in an upcoming issue of the *International Journal of Surgery*. This study is a bibliometric article, aiming to elucidate the changes, development trends, and research hotspots of neoadjuvant immunotherapy over the past few decades. On the whole, this study investigated the annual publication number, top contributors including authors, institutions and countries, active journals, as well as hotspot keywords in this field, which could provide an important reference for young academics and policymakers. As stated by the authors, neoadjuvant immunotherapy is gaining more and more attention for treating various types of cancer, this study is of great research significance. However, we have some concerns regarding the retrieval process which we would like to discuss with the authors.

Firstly, we agree with the authors that the Web of Science (WoS) could be the most appropriate database for bibliometric analysis. In this study, the relevant articles were identified in the Web of Science Core Collection (WoSCC) with all database versions. However, to our knowledge, WoSCC included at least 10 sub-databases including Science Citation Index Expanded (SCI-EXPANDED), Social Sciences Citation Index (SSCI), Arts & Humanities Citation Index (AHCI), Conference Proceedings Citation Index – Science (CPCI-S), Conference Proceedings Citation Index – Social Science & Humanities (CPCI-SSH), Book Citation Index – Science (BKCI-S), Book Citation Index – Social Sciences & Humanities (BKCI-SSH), Emerging Sources Citation Index (ESCI), Current Chemical Reactions (CCR-EXPANDED), and Index Chemicus (IC). In our opinion, it may not be appropriate to include all these sub-databases for searching eligible articles. For example, by using the retrieval formula from the authors, none of the related studies could be found in IC, CCR-EXPANDED, and AHCI. Consistent with this idea, some scholars also believe that it is unsuitable to use all these different types and levels of databases in one bibliometric analysis^2,3^. Among them, we and many previous studies suggest that SCI-EXPANDED could be the most appropriate database for performing bibliometric analysis.

Secondly, in this study, the author uses ‘TS’ as the field tag. According to WoS, TS refers to a topic search that comprises the title (TI), abstract (AB), author keywords (AK), and keyword plus (KP) terms. As for keywords, AK means keywords are provided by the authors, while KP are those automatically extracted by the system. In our experience, KP might not be appropriate to include in the search process. Although TS could expand the scope of the literature search, many unrelated kinds of literature in this field will also be included. For example, according to the method provided by the authors, we have summarized the top 20 highly cited studies from WoSCC in Table 1. After being manually screened, 40% of them are not related to neoadjuvant immunotherapy. Therefore, in order to minimize bias from the retrieval method, further optimization of the search strategy may be necessary.

**Table 1:** Top 20 highly cited studies on neoadjuvant immunotherapy.

Articles from Jiang *et al*.^1^	Related (YES/NO)
Neoadjuvant PD-1 Blockade in Resectable Lung Cancer	YES
Pembrolizumab for Early Triple-Negative Breast Cancer	YES
B cells and tertiary lymphoid structures promote immunotherapy response	YES
Low-Dose Irradiation Programs Macrophage Differentiation to an iNOS(+)/M1 Phenotype that Orchestrates Effective T Cell Immunotherapy	NO
Neoadjuvant anti-PD-1 immunotherapy promotes a survival benefit with intratumoral and systemic immune responses in recurrent glioblastoma	YES
Erdafitinib in Locally Advanced or Metastatic Urothelial Carcinoma	NO
Improved Efficacy of Neoadjuvant Compared to Adjuvant Immunotherapy to Eradicate Metastatic Disease	YES
Neoadjuvant immunotherapy leads to pathological responses in MMR-proficient and MMR-deficient early-stage colon cancers	YES
Neoadjuvant versus adjuvant ipilimumab plus nivolumab in macroscopic stage III melanoma	YES
RAS/MAPK Activation Is Associated with Reduced Tumor-Infiltrating Lymphocytes in Triple-Negative Breast Cancer: Therapeutic Cooperation Between MEK and PD-1/PD-L1 Immune Checkpoint Inhibitors	NO
Pembrolizumab as Neoadjuvant Therapy Before Radical Cystectomy in Patients With Muscle-Invasive Urothelial Bladder Carcinoma (PURE-01): An Open-Label, Single-Arm, Phase II Study	YES
Patterns of Immune Infiltration in Breast Cancer and Their Clinical Implications: A Gene-Expression-Based Retrospective Study	NO
ESMO-Magnitude of Clinical Benefit Scale version 1.1	NO
Immunotherapy Converts Nonimmunogenic Pancreatic Tumors into Immunogenic Foci of Immune Regulation	YES
A randomised phase II study investigating durvalumab in addition to an anthracycline taxane-based neoadjuvant therapy in early triple-negative breast cancer: clinical results and biomarker analysis of GeparNuevo study	YES
Treatment of muscle-invasive and advanced bladder cancer in 2020	NO
Quantitative Multiplex Immunohistochemistry Reveals Myeloid-Inflamed Tumor-Immune Complexity Associated with Poor Prognosis	YES
The Chinese Society of Clinical Oncology (CSCO): clinical guidelines for the diagnosis and treatment of gastric cancer	NO
PD-L1 Expression Correlates with Tumor-Infiltrating Lymphocytes and Response to Neoadjuvant Chemotherapy in Breast Cancer	YES
Predicting response to cancer immunotherapy using noninvasive radiomic biomarkers	NO

In addition, search terms are also very important because some medical terms represent the same thing although they have different forms. In this study, the author used the terms ‘Immunotherapy’ and ‘Immunotherapies’ to find immunotherapy-related datasets. We believe these terms could not totally identify all related studies and many potentially relevant papers may be missed. In our opinion, the author also should add the following terms into the search formula including ‘immune checkpoint inhibitors’, ‘immune checkpoint blockade’, ‘PD-L1’, ‘PD-1’, ‘CTLA-4’, and so on^4^. Moreover, many terms have plural and singular alternations. The author could use several wildcards such as ‘*’. The wildcard ‘*’ means it could be in place of any number of characters. For example, ‘Immunotherap*’ would also return the terms of ‘Immunotherapy’ and ‘Immunotherapies’.

Last but not least, as we all know, H-index refers to h articles in the literature that have been cited at least h times by other researchers, which is an important approach for estimating an author, institute, or journal by the academic output and level^5^. Therefore, it is not possible to obtain the result that the H-index is larger than the number of publications. However, as shown in Table 2 of this study (see^1^), the values of the H-index for authors far exceed the number of publications. Thus, the author should further explain how to obtain H-index in the method part. By the way, as Brigham and Women’s Hospital is affiliated with Harvard Medical School, thus it is probably more appropriate to merge them in Figure 4 (see^1^).

In sum, we congratulate the authors on this comprehensive bibliometric work organizing a large volume of data on this topic. Nevertheless, we also believe that our suggestions for the search process could help the authors acquire more reliable and accurate raw data for bibliometric analysis. Meanwhile, as more and more bibliometric studies are published in the biomedical area, we here call for a multicenter collaboration to create optimal guidelines for bibliometric studies.

## Ethical approval

This study does not include any individual-level data and thus does not require any ethical approval.

## Sources of funding

This study is supported by China Postdoctoral Science Foundation (2022M720385) and Beijing JST Research Funding (YGQ-202313).

## Conflicts of interest disclosure

The authors declare no conflicts of interest.

## Author contribution

K.C.: methodology, formal analysis, investigation, and writing – original draft; Y.H.: methodology, data curation, formal analysis, and resources; S.G.: methodology, data curation, formal analysis, and resources; H.W.: conceptualization, methodology, data curation, resources, and investigation; C.L.: conceptualization, formal analysis, and resources.

## Research registration unique identifying number (UIN)

1. Name of the registry: not applicable.
2. Unique identifying number or registration ID: not applicable.
3. Hyperlink to your specific registration (must be publicly accessible and will be checked): not applicable.

## Guarantor

Haiyang Wu and Cheng Li.
